# Primary Bile Duct Stones and Bacterial Activity

**DOI:** 10.1155/1992/81017

**Published:** 1992

**Authors:** Luis Vitetta, Avni Sali

**Affiliations:** Department of Surgery University of Melbourne Repatriation General Hospital Heidelberg Victoria 3081 Australia

## Abstract

The results of this study suggest that infection with beta-glucuronidase active bacteria is the initial event in the nucleation of primary bile duct stones (PBDS).

PBDS from five patients were morphologically fragile and “earthy” with alternating light and dark brown pigment layers with no evidence of a distinct central nucleus that may have been reminiscent of a different structure. Chemically, calcium bilirubinate and calcium palmitate were prominent throughout their structure. All bile duct biles had a positive culture and were always associated with at least one bacterial species which was beta-glucuronidase active. Moreover, fragments of PBDS nuclear areas had positive cultures that were comparable with those present in their individual bile duct bile. Microscopic examination of bile showed abundant precipitation of calcium bilirubinate granules in all samples.

Thus, bile duct bile infection with beta-glucuronidase active bacteria (e.g. *E. coli, C. perfringens*) appears to be a key factor in PBDS pathogenesis, having a precursor role, rather than being a consequence. Bile stasis is likely to be a co-factor which must have a supportive role in subsequent stone growth.


*HPB Surgery*, 1992, Vol. 6, pp. 23-33
Reprints available directly from the publisher
Photocopying permitted by license only
© 1992 Harwood Academic Publishers GmbH
Printed in the United Kingdom

### PRIMARY BILE DUCT STONES AND BACTERIAL ACTIVITY

LUIS VITETTA and AVNI SALI
*Department of Surgery, University of Melbourne, Repatriation General Hospital, Heidelberg, Australia*

*(Received 4 February 1992)*

The results of this study suggest that infection with beta-glucuronidase active bacteria is the initial event in the nucleation of primary bile duct stones (PBDS).

PBDS from five patients were morphologically fragile and "earthy" with alternating light and dark brown pigment layers with no evidence of a distinct central nucleus that may have been reminiscent of a different structure. Chemically, calcium bilirubinate and calcium palmitate were prominent throughout their structure. All bile duct biles had a positive culture and were always associated with at least one bacterial species which was beta-glucuronidase active. Moreover, fragments of PBDS nuclear areas had positive cultures that were comparable with those present in their individual bile duct bile. Microscopic examination of bile showed abundant precipitation of calcium bilirubinate granules in all samples.

Thus, bile duct bile infection with beta-glucuronidase active bacteria (e.g. *E. coli, C. perfringens*) appears to be a key factor in PBDS pathogenesis, having a precursor role, rather than being a consequence. Bile stasis is likely to be a co-factor which must have a supportive role in subsequent stone growth.

KEY WORDS: Aetiology, bacteria, nucleation, primary bile duct stones

### INTRODUCTION

Bile duct stones are classified as primary or secondary according to their site of origin. Primary bile duct stones (PBDS) or brown pigment bile duct stones as they are alternatively known, are those that form in the bile duct, whereas secondary bile duct stones (SBDS) are stones that form in the gallbladder and which later migrate into the bile duct[1 2].

PBDS are classified as brown pigment bile duct stones, soft, with alternating light and dark brown pigment layers on cross-section. Chemically they contain low levels of cholesterol, with high levels of bilirubin and calcium. Infrared spectroscopy studies have shown that calcium is present in the form of calcium bilirubinate and calcium palmitate[3-6].

The pathogenesis of PBDS has remained largely elusive until recent times. The pigment component of brown stones is documented as originating from deconjugated bilirubin[7]. Since infection was a common finding with brown stones, Maki postulated that bacterial beta-glucuronidase deconjugated bilirubin diglucuronide to unconjugated bilirubin[7]. This free bilirubin was then capable of binding ionised

Address correspondence to: Dr L. Vitetta, University Department of Surgery, Repatriation General Hospital, Heidelberg 3081, Victoria, Australia calcium which leads to calcium bilirubinate (CaB) precipitation and stone initiation. Recently Cetta further hypothesised that calcium palmitate in brown recurrent bile duct stones may also originate from the action of a bacterial phospholipase[3]. He has thus suggested that bile infection precedes brown pigment stone formation in at least some patients.

The clinical and laboratory findings from eight patients with PBDS recurrent at least 18 months after cholecystectomy are presented. PBDS composition, and bacteriology were related to bile duct bacteriology and microscopy studies. The results suggest that bacterial infection precedes PBDS formation, and that bacterial beta-glucuronidase activity is a key factor in the initiation and growth of PBDS.

### PATIENTS AND METHODS

### *Patients Studied*

Eight patients with bile duct stones recurrent at least 18 months after cholecystectomy were studied (Table 1).

**Table 1** Patient Characteristics at Cholecystectomy

| *Patient* | *A* | *B* | *C* | *D* | *E* | *F* | *G* | *H* |
|---|---|---|---|---|---|---|---|---|
| Present Age (Years) | 56 | 45 | 55 | 69 | 40 | 42 | 45 | 52 |
| Sex | F | F | F | F | F | M | F | M |
| Previous Cholecysectomy (Years): | 4 | 11 | 12 | 40 | 2 | 2 | 4 | 5 |
| Gallbladder Stones Type: | +BP | BP | MC | ? | MC | MC | MC | MC |
| Gallbladder/Bile Culture: | ? | ? | −ve | ? | +ve | −ve | −ve | −ve |
| Bile Ducts: | NAD | NAD | NAD | NAD | Stones | *NAD | NAD | NAD |

**Note:** * At Cholecystectomy gallstones were recovered from the gallbladder, cystic duct and bile duct. +BP = Black Pigment; MC = Multiple Cholesterol; NAD = No Abnormalities Detected.

*Patients A and B*: Cholecystectomies were performed for gallbladder disease with black pigment gallstones. Initial operations were performed in Europe, hence a detailed outline of their case reports and pathology results were not known. They presented to us with symptoms of obstructive jaundice. Bile duct stones and bile duct bile were obtained from each patient at a second operation that consisted of an operative cholangiogram, hepaticojejunostomy, and repair of an incisional hernia for patient A, and an operative cholangiogram for patient B.

*Patients C, F, G and H*: Cholecystectomies and operative cholangiograms were performed on each patient for gallbladder disease with multiple cholesterol gallstones. Their respective bile ducts at the time of cholecystectomy were normal with no abnormalities detected. Gallbladder bile cultures were negative in each case. Recovery was uneventful. At least two years after cholecystectomy, each patient developed a new episode of biliary colic with associated chills and fever. Endoscopic retrograde cholangiopancreatography (ERCP) showed single bile duct stones in patients G and H. At operation, bile duct stones and bile were recovered from patients C, F and G only.

*Patient D*: This patient had a long history of recurrent abdominal pain. A cholecystectomy had been performed 40 years previously. Since then the patient had undergone three choledochoduodenostomies and reversal of these in 1978. This patient's latest episode of pain (1988) comprised a severe episode of right upper quadrant pain. Ultrasonographic investigations demonstrated multiple bile duct stones. The patient was admitted for ERCP and sphincterotomy. At ERCP three bile duct stones were removed.

*Patient E*: This patient originally underwent a cholecystectomy and operative cholangiogram for acute cholecystitis. Cholesterol gallstones were removed from the gallbladder, cystic duct and bile duct, and gallbladder bile culture was positive for bacteria that were beta-glucuronidase negative. Approximately one year later the patient was readmitted with pain and jaundice. A clinical diagnosis of obstructive jaundice was made. Following two successful ERCP investigations four weeks apart, no calculi were visualised, but a considerable amount of purulent material was observed to drain from the ampulla on each occasion. Six months later the patient was readmitted to hospital with bile duct stones in a dilated bile duct. Bile duct obstruction due to primary bile duct stones was diagnosed at ERCP on this occasion. Stones were unavailable for clinical analysis.

### *Bile Duct Stone Morphology*

Bile duct stones were examined for gross morphology by noting surface features, texture and cross-sectional appearance (Figure 1). The distribution of cholesterol and pigment was noted. Biliary sludge was obtained from three patients (Figure 2).

### *Bile Duct Stone Chemistry*

Bile duct stones were analysed via chemical analysis and infrared spectroscopy[8–14]. All reagents for the chemical analysis and infrared spectroscopy were of analytical and spectral grade respectively. Cholesterol, calcium, bilirubin and total calcium were quantitated via chemical analysis[8–11].

Infrared spectroscopy was used to detect the presence of common PBDS components, namely cholesterol, calcium bilirubinate and calcium palmitate. Briefly, 1 mg aliquots of dessicated stone powder were finely ground with an agate mortar and pestle, and then mixed and reground with 100 mg of spectral grade potassium bromide. The resulting fine homogenous powder mix was placed in a stainless steel die and pressed at 25 000 psi in a hydraulic press for five minutes. An infrared spectrum was obtained on the wafer with a Perkin-Elmer 457 grating infrared spectrophotometer. Reference infrared specta of cholesterol (Sigma), and calcium bilirubinate and calcium palmitate (prepared according to previously described methods) were also recorded[12,13,14].

### *Bile Duct Bile and Stone Bactibilia*

Bile duct samples were examined for bacteria by Gram's stain. Biles were directly plated onto sheep blood agar (SBS), McConkey's agar, horse blood agar (HBA), lysed horse blood with Vancomycin and vitamin K (LKV), Nagler's agar, phenylethyl alcohol agar, and cooked meat medium. HBA, LKV, Nagler, phenylethyl alcohol agar and cooked meat medium were incubated anaerobically in jars flushed with a commercial gas mixture (85%$N_2$ 5%$H_2$ 10%$Co_2$). All plates were incubated at 35° C for five days and examined daily for growth. Bacterial growth was graded as a function of colony forming units (CFU) per millilitre of bile or bile duct stone fragment-nutrient media suspension. Scant growth was a CFU/ml of less than 1000, moderate 10,000–100,000 CFU/ml and profuse growth a CFU/ml of greater than 100,000.

Since the bile duct stones recovered were extremely fragile our attempt to culture the central area completely separate from the rest of the stone was not possible. However, a nuclear fragment of those bile duct stones that were available was washed with saline, carefully avoiding further fragmentation, and then suspended in 5 ml of nutrient media. The suspension was then plated and incubated as previously described and the plates examined daily for growth.

### *Bacterial Beta-glucuronidase Activity*

To detect the activity of bacterial beta-glucuronidase, all bacterial species isolated from the bile ducts and stones were subcultured and the enzyme qualitatively estimated by the previously described method of Tabata and Nakayama[15].

Briefly the hydrolytic activity of beta-glucuronidase was determined by incubating the bacteria, previously grown in a nutrient broth, with a substrate, beta-nitrophenyl-beta-D-glucuronic acid for 1–4 hours at 37 °C. A positive test for beta-glucuronidase activity was indicated by the development of yellow colour.

### *Microscopy of Bile Duct Biles*

Bile samples from the common bile duct were examined for biliary crystals (cholesterol (ChC), calcium bilirubinate (CaB)) within five minutes of aspiration (Figure 3). Biles were mixed thoroughly and placed on glass slides and examined microscopically in direct and polarised light at low (×100) and high (×400) magnifications[16].

Bile duct biles were examined microscopically to detect CaB granules, the presence of which was confirmed by comparison with synthetic CaB samples synthesised in our own laboratory by the methods of Edwards and colleagues[17], as well as Sutor and Wilkie[13] and chemically verified by infrared spectroscopy. As previously described by Juniper and Buson CaB pigment granules occur in bile as yellowish-brown clumps[18].

### RESULTS

### *Bile Duct Stones*

Bile duct stones were recovered from five of eight patients with choledocholithiasis. All of the stones were dark brown in colour and ovoid in shape with contours that adhered to the shape of the bile duct. They were fragile, and could easily be crushed between the fingers. On cross-section all stones presented with alternating light and dark brown layers, with no evidence of a distinct central nucleus that may have represented a different structure (Figure 1).

The composition of the bile duct stones showed that they contained high levels of bilirubin and calcium but low levels of cholesterol. Infrared spectroscopy showed that all samples contained cholesterol and calcium in the form of the salts of bilirubinate and palmitate (Table 2).

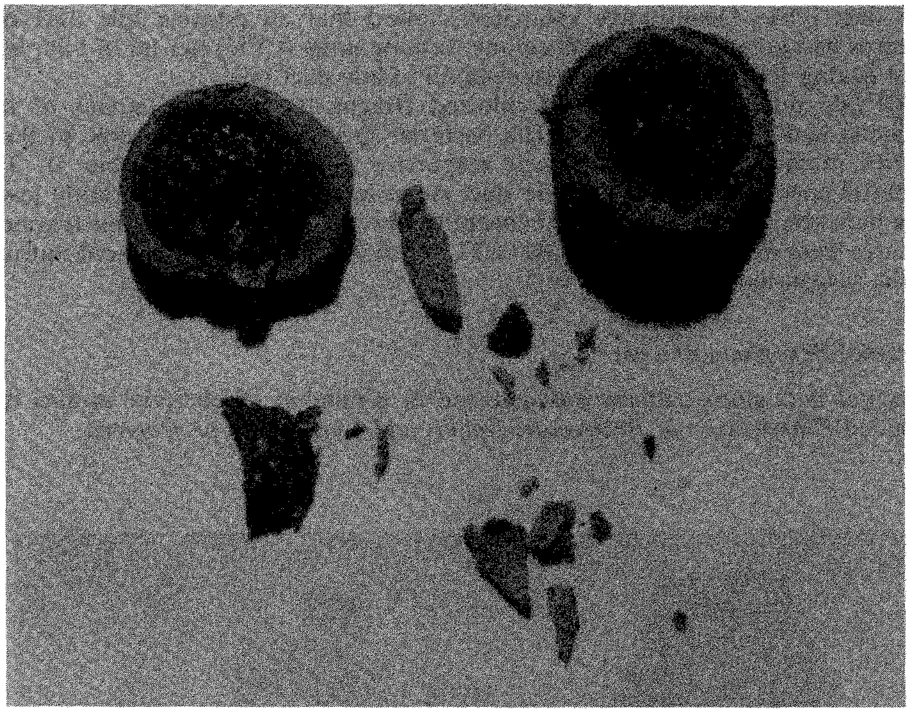

**Figure 1** Primary bile duct stone sample (Patient C).

**Table 2** PBDS Characteristics

| *Patient* | *A* | *B* | *C* | *D** | *E** | *F* | *G* | *H** |
|---|---|---|---|---|---|---|---|---|
| PBDS | M | M | S | S | M | S | S | S |
| and | | | | | | | | |
| Sludge | + | – | – | + | + | – | – | – |
| PBDS | | | Composition % Dry Weight | | | | | |
| Cholesterol: | 29 | 31 | 34 | | | 23 | 32 | |
| Bilirubin: | 28 | 20 | 25 | | | 22 | 19 | |
| Calcium: | 10 | 10 | 5 | | | 9 | 12 | |
| IR Spectroscopy | | | | | | | | |
| Calcium Bilirubinate: | | | | | | | | |
| Nuclear Area: | + | + | + | | | + | + | |
| Peripheral Area: | + | + | + | | | + | + | |
| Calcium Palmitate: | | | | | | | | |
| Nuclear Area: | + | + | + | | | + | + | |
| Peripheral Area: | + | + | + | | | + | + | |

*Note:* *Bile duct stones unavailable/lost at endoscopy. (M) Multiples (S) Single.

*Bile Duct Bile and Stone Bactibilia*

Positive bile cultures occurred in all the bile duct samples. Six of eight bile duct biles were associated with more than one bacterial strain. A number of aerobic and anaerobic bacterial species were isolated from the positive bile cultures. The most common bacterial species isolated was *E. coli* (Table 3). It was isolated in 5/8 of the bile duct biles. Quantitative estimates showed that all positive bile duct bile cultures had greater than 10,000 colony forming units (CFU) per ml of bile, with most having 100,000 CFU or greater per ml of bile duct bile.

All bile duct stone fragments cultured (fragments from the stones of five patients) had a positive culture. The bacterial species were comparable to those species previously cultured in their respective bile duct biles (Table 3). The bacterial concentrations of the bile duct stone fragment were significantly lower than their respective bile duct bile concentrations. Positive bile duct stone fragment cultures were no greater than 10,000 CFU/ml of bile duct stone fragment-nutrient media suspension.

*Bacterial Beta-glucuronidase*

Bacterial beta-glucuronidase was detected in a number of bacterial strains that included *Esherichia coli, Bacteroides fragilis* and *Clostridium perfringens*.

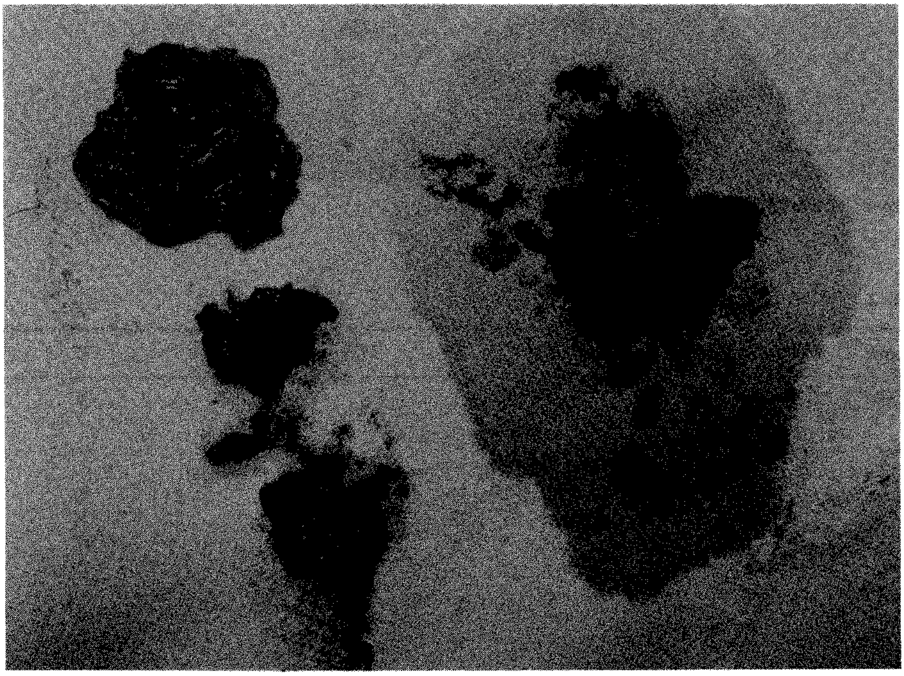

**Figure 2** Bile duct sludge sample (Patient A).

**Table 3** Bactibilia and Microscopy of Bile Duct Bile

| *Patient* | *A* | *B* | *C* | *F* | *G* |
|---|---|---|---|---|---|
| BD Bile/Bacteria + CFU | E. Coli$^{3}$<br>S. Faecalis$^{3}$<br>K. Oxytoca$^{3}$ | B. Fragilis$^{3}$ | E. Coli$^{1}$<br>K. Pneumoniae$^{1}$<br>C. Perfringens$^{3}$ | E. Coli$^{3}$<br>K. Pneumoniae$^{2}$ | C. Perfringens$^{3}$<br>K. Oxytoca$^{2}$<br>E. Coli$^{2}$ |
| PBDS Fragments/Bacteria + CFU | E. Coli$^{1}$<br>S. Faecalis$^{1}$<br>K. Oxytoca$^{1}$ | B. Fragilis$^{1}$ | E. Coli$^{1}$<br>C. Perfringens$^{1}$ | E. Coli$^{1}$<br>K. Pneumoniae | C. Perfringens$^{1}$<br>K. Oxytoca$^{2}$<br>E. Coli$^{1}$ |
| Microscopy BD Bile/Chc | + | + | − | + | − |
| Microscopy BD Bile/CaB | + | + | + | + | + |

Note: *1 = $10^3 - 10^4$ $CFU/_{ML}$ 2 = $10^4 - 10^5$ $CFU/_{ML}$ 3 = > $10^5$ $CFU/_{ML}$

*Microscopy of Bile Duct Biles*

When bile duct bile was observed under direct and polarised light at low magnification power ( × 100) calcium bilirubinate granules were readily observed in all samples (Figure 3). Cholesterol crystals were not easily observed in any sample at low power. However, at a higher power of magnification ( × 400) under polarised light, a few cholesterol crystals were observed in 3/5 of the bile duct bile samples (Table 3).

**Figure 3** Microscopy of bile duct bile (magnification × 100) showing abundant CaB precipitation (Patient B). (*See colour plate I at back of issue*)

### DISCUSSION

In this present study, bile duct stones were recovered from five of the eight patients, and their morphological features and chemical compositions indicated that they were consistent with PBDS previously described[1,2,4]. Brown pigment, fragile "earthy" stones were observed and on cross-section presented with a pigmented layered appearance with diffuse nuclear areas that were not reminiscent of gallbladder stones that were previously present in these patients. Indeed, six patients were noted to have had gallbladders with multiple cholesterol and two with black pigment gallstones at the time of cholecystectomy. Thus the bile duct stones were almost certainly formed *de novo* in the bile ducts.

When our group first described the likely sequence of events leading to PBDS formation resulting from bile duct infection and subsequent CaB precipitation[5], it was not clearly known whether the initiation of PBDS preceded or followed pathophysiological changes in the bile duct and/or its environment. It was known that the formation of stones in the bile duct was associated with stasis and bile duct strictures (traumatic and non-traumatic). Frequently, dilated ducts were present[19], thus giving the impression that PBDS formation was the result of some "trigger" factor that brought about bile stasis. Bile stasis was thought to lead to stone formation within the biliary tract and infection being caused by the stones.

The present study with Australian patients (of European descent) alternatively suggests that it is the stones themselves that are the consequence of bile duct bile infection, thus forming *de novo* in the bile ducts. Cetta recently showed in a prospective study of 600 patients who underwent operation for gallstones, that infection preceded PBDS formation[3]. The results from two patients indicated that the precursor event in stone formation was indeed of bacterial origin[3].

Although it was fundamental to first establish that the bile duct stones were PBDS, a most important question was whether bile duct bile infection had anticipated stone formation in the bile ducts. As far as could be deduced from the patients' previous gallbladder operations and medical histories, most of the patients studied (seven of eight) showed no apparent biliary infection at the time of cholecystectomy or on leaving hospital. However, the observation of bacteria present within fragments of all PBDS studied, as well as their chemical composition, strongly suggested that the bacteria may have preceded PBDS formation.

A key factor in this study was the presence in all bile duct bile samples tested, of at least one glucuronidase-producing bacterial strain in profuse growth concentrations. This in part supported the hypothesis proposed by Maki[7], that bacterial beta-glucuronidase if present may hydrolyse bilirubin conjugates in bile, thereby promoting calcium bilirubinate precipitation. In a recent study by Skar and colleagues[20], it was shown that beta-glucuronidase activity was related to bacterial growth in common bile duct biles of gallstone patients. They hence showed that patients with beta-glucuronidase producing bacteria had on average a significantly higher enzyme activity in bile than patients without such bacteria.

Thus the combined results presented in this study link together a number of observations that support our "infection first" hypothesis, namely, the occurrence of at least one bacterial species with a positive beta-glucuronidase activity, abundant calcium bilirubinate precipitation (Figure 3), and PBDS "rich" in CaB, all concomitantly present in bile duct bile. Moreover, those bile duct stone core fragments that were available had a positive culture for bacterial species that were exactly comparable to those species isolated from their respective bile duct bile samples, a result that further supported our hypothesis.

Cetta[3] has proposed that bacterial phospholipase may also provide the catalyst action for the precipitation of calcium palmitate, a common component of PBDS. We have always shown the presence of calcium palmitate in PBDS[5,22]. Calcium palmitate has also been observed in some gallstones that were associated with gallbladder bile infection[21]. Hence, it is likely that specific bacteria have a central role in PBDS formation because of their association with the pigment and fatty acid components of these stones.

Bile stasis is likely to have a supportive role secondary to infection. It has been shown that there is abundant CaB precipitation in the bile of patients with PBDS. The CaB precipitation in bile could impede the normal bile duct flow, resulting in bile stasis. The presence of mechanical aberrations of the bile duct such as stricture, cystic duct stump, suture material or duodenal diverticula near the ampulla could further enhance the stasis problem. Therefore the combined results of this study strongly suggest that the precursor event in PBDS formation and growth may be intimately associated with infection of bile duct bile, specifically with beta-glucuronidase active bacteria.

### *References*

1. Bernhoft, R.A., Pellegrini, C.A., Motson, R.W. and Way, L.W. (1984) Composition and morphologic and clinical features of common duct stones. *Am. J. Surg.*, **148**, 77–85
2. Whiting, M.J. and Watts, J.McK. (1986) Chemical composition of common bile duct stones. *Br. J. Surg.*, **73**, 229–232
3. Cetta, F.M. (1986) Bile infection documented as initial event in the pathogenesis of brown pigment biliary stones. *Hepatology*, **6**, 482–489
4. Nayman, A.J., Vitetta, L., Sali, A. and Hocking, C. (1986) Primary bile duct stones. *Aust. NZ J. Surg.*, **52**, 280–281
5. Vitetta, L., Sali, A. and Nayman, J. (1986) Is the aetiology of primary choledocholithiasis unique? *Dig. Surg.*, **3**, 126
6. Masuda, H. and Nakayama, F. (1979) Composition of bile pigment in gallstones and bile and their etiological significance. *J. Lab. Clin. Med.*, **83**, 353–360
7. Maki, T. (1966) Pathogenesis of calcium bilirubinate gallstones. *Ann. Surg.*, **164**, 90–100
8. Roschlau, P. (1974) Test combination cholesterol. Catalase method (Boehringer-Mannheim). *Z. Klin. Chem. Klin. Biochem.*, **12**, 403
9. Malloy, H.T. and Evelyn, K.H. (1936) The determination of bilirubin with the photoelectric colorimeter. *J. Biol. Chem.*, 119–481
10. Henry, R.H., Cannon, D.C. and Winkelman, J.W. (1974) In: *Clinical Chemistry Principles and Techniques*, Ch 22, p. 1051. New York: Harper and Row
11. Nakayama, F. (1968) Quantitative microanalysis of gallstones. *J. Lab. Clin. Med.*, **72**, 602–611
12. Trotman, B.W., Morris, T.A., Sandex, H.M. *et al.* (1977) Pigment versus cholesterol cholelithiasis: Identification and quantification by infrared spectroscopy. *Gastroenterol*, **72**, 495–498
13. Sutor, D.J. and Wilkie, L.I. (1977) The crystalline salts of calcium bilirubinate in human gallstones. *Clin. Sci. Mol. Med.*, **53**, 101–103
14. Henichart, J.P. and Bernier, J.L. (1982) Identification of calcium palmitate in gallstones by IR spectroscopy. *Clin. Chim. Acta.*, **118**, 279–287
15. Tabata, M. and Nakayama, F. (1980) Bacteria and gallstones -- etiological significance. *Dig. Dis. Sci.*, **26**, 218–244
16. Sedaghat, A. and Grundy, S.M. (1980) Cholesterol crystals and the formation of cholesterol gallstones. *New. Eng. J. Med.*, **302**, 1274–1277
17. Edwards, J.D., Adams, W.D. and Halpert, B. (1958) Infrared spectrum of biliary calculi. *Am. J. Clin. Path.*, **29**, 236–239
18. Juniper, K.M. and Burson, E.N. (1954) A note on the incidence of cholelithiasis. *Gastroenterol.*, **32**, 175–211
19. Meshkinpour, H., Schapiro, M. and Mason, G.R. (1985) Choledocholithiasis. In: *Bockus Gastroenterelogy*, 4th ed, Vol. 6, Ch. 193, p. 3693. New York: W.B. Saunders
20. Skar, V., Skar, A.G. and Stromme, J.H. (1988) Beta-glucuronidase activity related to bacterial growth in common bile duct bile in gallstone patients. *Scand. J. Gastroent.*, **23**, 83–90
21. Vitetta, L., Sali, A., Moritz, V., Shaw, A., Carson, P., Little, P. and Elzarka, A. (1989) Bacteria and gallstone nucleation. *Aust. NZ J. Surg.*, **59**, 571–577
22. Vitetta, L., Sali, A., Little, P., Nayman, J. and Elzarka, A. (1991) Primary "brown pigment" bile duct stones. *HPB Surgery*, **4**, 209–222

(*Accepted by M. Little 23 January 1992*)

### INVITED COMMENTARY

Ever since Maki[1] first suggested that infection with beta glucuronidase secreting organisms might deconjugate bilirubin diglucuronide to unconjugated bilirubin, producing primary bile duct stones, the hypothesis has been widely quoted without clear confirmation. The more recent suggestion by Cetta[2] that calcium palmitate might be precipitated by the action of bacterial phospholipase has added some theoretical credibility to the postulate that biliary infection might precede primary bile duct stone formation[3], rather than follow the formation of stones. Work from Nakayama and his group[4,5] has also lent weight to the infective theory of bile duct stone formation.

The present paper reports eight patients with what are presumed to be primary bile duct stones. In five, the morphological characteristics of the stones seem appropriate for that diagnosis, and the clinical features of the other three are certainly consistent with the diagnosis of primary bile duct stones. The authors are to be congratulated on the thoroughness with which they have investigated their patients, and it is of considerable significance that beta glucuronidase secreting bacteria were recovered from the bile of all patients. The recovery of beta glucuronidase secreting organisms from the nuclear region of stones is perhaps a little less convincing, given the friability of the stones and the difficulty of identifying the nuclear regions. Nevertheless, Vitetta and Sali have made a significant contribution to the data which supports the role of beta glucuronidase secreting organism in the genesis of primary bile duct stones.

### *References*

1. Maki, T. (1966) Pathogenesis of calcium bilirubinate stones; role of E. coli, beta glucuronidase and coagulation by inorganic ions, polyelectrolytes and agitation. *Ann. Surg.*, **164**, 90–100
2. Cetta, F.M. (1986) Bile infection documented as the initial event in the pathogenesis of brown pigment stones. *Hepatology*, **6**, 482–489
3. Feratis, Ch.B., Contour, C.Th., Manouras, A.J., Apostolidis, N.S., Golematis, B.Ch. (1984) Long term consequences of bacterial colonization of the biliary tract after choledochochostomy. *Surg. Gynec. Obstet.*, **159**, 363–366
4. Masuda, H. and Nakayama, F. (1979) Composition of bile pigment in gall stones and bile and their etiological significance. *J. Lab. Clin. Med.*, **83**, 353–360
5. Tabata, M. and Nakayama, F. (1980) Bacteria and gallstones -- etiological significance. *Dig. Dis. Sci.*, **26**, 218–244

J.M. Little
Department of Surgery
Westmead Hospital
Westmead N.S.W. 2145
Australia

